# The Cessation of the Elimination of Odors a Sign of Bright’s Disease

**Published:** 1859-03

**Authors:** 


### ECLECTIC DEPARTMENT,

### AND SPIRIT OF THE MEDICAL PERIODICAL PRESS.

---

*The Cessation of the Elimination of Odors a sign of Bright's Disease.*— M. De Beauvais read a paper on the "Deficent Elimination of Odorous Substances through the Urine in Bright's Disease," at the meeting of the Academy of Sciences, October 25th, from which we take the following conclusions:

"Odorous substances, fixed or volatile, do not pass by the urine in confirmed cases of Bright's disease, so long as the coloring matters are eliminated. Since 1849 I have continued my experiments with the juice of asparagus, or with the essence of turpentine. I have repeated them, without interruption, on a great number of subjects, at different stages of albuminuria, in the service of Prof. Rostan, during my residence as *interne* in Hotel Dieu, in 1854, '55, and 56. In the convulsions of children, as in those of pregnant and lying-in-women; in scarlatina complicated with anasarca; in diseases of the brain; in neuroses; in paraplegia with lesion of the genito-urinary organs; in organic affections of the heart, liver, lungs, kidneys; in purpura, scurvy, diabetes, fevers, phlegmasiæ, diseases of the skin; in the principal cachexiæ, and cholera, I have easily determined, by the aid of this particular sign, if albuminuria was connected with the existence of lesions belonging to Bright's disease. Indeed, I repeat the fact that the suppression of the function of eliminating odors does not take place, except in this affection exclusively. It is constant, absolute, incurable. The following example demonstrates this:

"In a man attacked with Bright's disease, whom I treated for five years, I never saw the passage of odors reäppear in the urine, in spite of the general dropsy and the notable diminution of the albumen, and the real amendment of the constitution.

"*Deductions.*—Albuminuria may, then, in these cases, cease for a longer or shorter time, but the passage of odors is never reëstablished—a capital fact, which demonstrates the persistence of the lesions, and the impossibility of the radical cure of Bright's disease. The autopsies made at Hotel-Dieu sustain us in stating that this functional trouble coincides almost always with anatomical lesions of the second stage of Bright's disease. In a pathological view, the suppression of this curious function, observed exclusively in Bright's disease, proves the speciality of this affection, and the morbid changes which are peculiar to it. In a physiological view, this abolition of elimination of odors confirms the importance and the nature of the *rôle* of the cortical substance, in the secretion and elaboration of the urine. I regard to prognosis and therapeutics, this particular sign reveals at once the gravity and fatal incurability of the confirmed disease.

"*Conclusions.*—With these premises, I lay down the three following propositions: 1st. The deficiency in the elimination of odorous substances by the urine is an exclusive sign pathognomonic of Bright's disease. 2d. This new sign, well ascertained, confirms, at the first view, the value of the symptom, albuminuria, the degree and the nature of the corresponding anatomical lesion. 3d. In default of albuminuria, a capital symptom, or of characteristic dropsy, the absolute suppression, incurable from the passage of odors in the urine, imposes on us at once the diagnosis, prognosis and treatment.—*Cincinnati Lancet and Observer.*

